# Vision-related quality of life in adults with severe peripheral vision loss: a qualitative interview study

**DOI:** 10.1186/s41687-020-00281-y

**Published:** 2021-01-13

**Authors:** Ryan Lange, Abigail Kumagai, Sara Weiss, Katherine B. Zaffke, Sherry Day, Donna Wicker, Ashley Howson, K. Thiran Jayasundera, Lori Smolinski, Christina Hedlich, Paul P. Lee, Robert W. Massof, Joan A. Stelmack, Noelle E. Carlozzi, Joshua R. Ehrlich

**Affiliations:** 1 Department of Ophthalmology and Visual Sciences, Center for Eye Policy and Innovation, University of Michigan, 1000 Wall Street, Ann Arbor, MI 48105 USA; 2 Association for the Blind and Visually Impaired, Grand Rapids, MI USA; 3 Institute for Healthcare Policy and Innovation, University of Michigan, Ann Arbor, MI USA; 4 Department of Ophthalmology, Johns Hopkins University, Baltimore, MD USA; 5 Edward Hines, Jr. VA Hospital, Hines, IL USA; 6 Department of Physical Medicine and Rehabilitation, University of Michigan, Ann Arbor, MI USA; 7 Center for Outcomes Development and Application, University of Michigan, Ann Arbor, MI USA

**Keywords:** Vision-related quality of life, Glaucoma, Retinitis pigmentosa, Peripheral field loss, Peripheral vision, Low vision, Vision rehabilitation, Patient-reported outcome, Qualitative research, Interview

## Abstract

**Background:**

Existing patient-reported outcome (PRO) measures may not be relevant to the full range of functional and vision-related quality of life (VR-QOL) concerns of individuals with vision impairment due to severe peripheral field loss (PFL). Measurement of VR-QOL in severe PFL is important in order to determine the effectiveness of vision rehabilitation interventions for this population. The purpose of this study was to characterize the impact of severe PFL due to retinitis pigmentosa (RP) and glaucoma on VR-QOL as the initial phase in the development of a novel PRO measure.

**Methods:**

Individuals with severe PFL due to RP or glaucoma were recruited from the Kellogg Eye Center and the Association for the Blind and Visually Impaired. Participants completed semi-structured qualitative interviews, the Impact of Vision Impairment (IVI) questionnaire and the RAND 36-Item Health Survey. Interviews were analyzed by two coders using thematic analysis. A matrix analysis was conducted to compare VR-QOL by cause of severe PFL. Sample size was determined by thematic saturation.

**Results:**

The study included 37 participants (19 RP, 18 glaucoma). Median best-corrected visual acuity for those with RP and glaucoma was 20/40 and 20/27.5, while Pelli-Robson contrast sensitivity was 1.2 log contrast sensitivity (logCS) and 1.1 logCS, respectively. Median domain scores on the IVI (reading, mobility, well-being) ranged from a low of − 0.2 to a high of 0.7 logits in those with RP and from 0.5 to 1.2 logits in those with glaucoma. Qualitative interviews identified six VR-QOL themes relevant across participants with both RP and glaucoma, including activity limitations, driving, emotional well-being, reading, mobility, and social function. VR-QOL concerns were largely consistent among those with severe PFL due to RP and glaucoma. These overarching themes contained content relevant to specific challenges related to severe PFL.

**Conclusions:**

There are commonly occurring VR-QOL concerns among individuals with severe PFL due to RP and glaucoma. The outlined themes will serve as the basis for development of the Low Vision Severely Constricted Peripheral Eyesight (LV-SCOPE) Questionnaire.

**Supplementary Information:**

The online version contains supplementary material available at 10.1186/s41687-020-00281-y.

## Background

Over the coming decades, the number of adults with blindness and visual impairment in the U.S. is predicted to double and the cost of treating vision loss is expected to increase from $145 billion to $376 billion [1, 2]. When blindness and visual impairment are chronic, uncorrectable and impact daily life, they may be termed *low vision* [3]. Among patients seen in clinical low vision practices, up to 85% may experience improved vision-dependent functioning through low vision rehabilitation [4, 5]. Despite the potential benefits of vision rehabilitation, there has been limited research in this area. Notably, no randomized controlled trial of vision rehabilitation [6] has targeted patients with peripheral field loss (PFL), a recognized cause of impaired functioning [7–9] and decreased vision-related quality of life (VR-QOL) [10, 11] that often results from conditions such as retinitis pigmentosa (RP) and glaucoma.

Although the prevalence of low vision due to PFL is not fully known, a survey of vision rehabilitation centers across the U.S. found that 21% of patients had a diagnosis of glaucoma or retinal degeneration [12], and in a multicenter study Goldstein et al. determined that 14% of patients presenting for vision rehabilitation had one of these diagnoses [13]. However, these data may underestimate the true burden of PFL since conditions such as non-glaucomatous optic neuropathies or a history of panretinal photocoagulation (e.g., for diabetic retinopathy) may also cause PFL, but do so less consistently or were not sufficiently captured. Prior investigations on the impact of severe PFL have focused largely on mobility [14–19], a dimension of VR-QOL that is prominently affected in PFL. However, there is also evidence that PFL can affect other domains of VR-QOL [20–22], though a multi-dimensional instrument to measure the impact of severe PFL on VR-QOL does not yet exist.

Since low vision services are tailored to meet the rehabilitation goals of individual patients, outcome measures to assess the effectiveness of vision rehabilitation are often patient-centered, in the form of patient-reported outcome (PRO) or performance-based outcome measures [23, 24]. However, PROs used in low vision were developed and validated among populations with primarily central vision loss, and investigators have noted problems, including poor targeting of items and inadequate content validity, when applying these outcomes to individuals with PFL [14, 25–31]. One possible explanation is that there are distinct impairments caused by peripheral and central vision loss [32]. Furthermore, older PROs may not include content relevant to common current technologies and mobility solutions. Accordingly, a contemporary, valid, and reliable PRO built around the VR-QOL and functional challenges of individuals with severe PFL may serve as a valuable measurement tool for future clinical trials and comparative effectiveness research to measure the effect of vision rehabilitation in this population.

While prior qualitative studies on VR-QOL in RP and glaucoma have been published [32–35], there is little data specific to how severe PFL affects VR-QOL. The current study provides a qualitative analysis of the impact of severe PFL on VR-QOL. This investigation is the first phase in the development of the Low Vision Severely Constricted Peripheral Eyesight (LV-SCOPE) Questionnaire, which will be a novel PRO developed to measure vision-related functioning in individuals with severe PFL with the goal of fostering future research on the effectiveness of vision rehabilitation in this population.

## Materials and methods

### Participants

Participants were recruited from the Kellogg Eye Center at the University of Michigan and the Association for the Blind and Visually Impaired (Grand Rapids, Michigan). A purposive sampling strategy was used to achieve a balance of age and gender and to oversample those with preserved visual acuity (defined as mild VI or better, ≥ 20/60). All participants met the U.S. definition of statutory blindness [36] and self-identified as having functional difficulty due to their vision loss (answered “yes” to the question “Does your vision loss impact your day-to-day life?”).

- Inclusion criteria for RP: RP diagnosed by a retina specialist and widest diameter ≤ 20 degrees on III4e isopter of the Goldmann visual field (GVF) in each eye (or in only one eye if the fellow eye visual acuity was < 20/200).
- Inclusion criteria for glaucoma: glaucoma diagnosed by a glaucoma specialist; reliable (< 33% false-positives, false-negatives, and fixation losses) and repeatable Humphrey (24–2 or 30–2 algorithm) visual field defect in better-seeing eye (defined based on visual acuity) classified as mixed or generalized-type Stage 4 or Stage 5 using the Enhanced Glaucoma Staging System [37].
- Exclusion criteria were: diagnosed cognitive impairment; Mini-Mental State Exam score ≤ 23; diagnosed severe mental illness; need for an interpreter; or a physical disability that prevented independent ambulation.

Participants with Usher Syndrome were eligible for inclusion in order to maintain generalizability to individuals with RP syndromes. Written informed consent was obtained from all participants. This study was approved by the University of Michigan Institutional Review Board and the research adhered to the tenets of the Declaration of Helsinki.

Trained research assistants (AK and KBZ) screened medical records to select participants who were likely to meet study criteria. For eligible participants, best-corrected Snellen visual acuity (BCVA) and results of visual field testing from within the prior 12 months were extracted from the chart. At the time of study enrollment, participants completed binocular Pelli-Robson contrast sensitivity testing, the Impact of Vision Impairment (IVI) questionnaire [38, 39], and the RAND 36-Item Health Survey (SF-36) [40]. Additional data were collected on educational attainment, employment status, living situation, age of diagnosis, medical comorbidities, and prior low vision interventions.

### Interviews

A semi-structured interview guide was developed based on a review of the existing literature (Supplementary Appendix). Questions were open-ended, with frequent probes to clarify, encourage, and elaborate. Participants were instructed to answer all questions based on their usual use of any adaptations, low vision devices, white cane, or guide dog. Interviews were conducted from November 2017 to January 2019 at the Kellogg Eye Center and the Association for the Blind and Visually Impaired. Two authors (AK and JRE) performed the interviews in person, which were audio recorded and transcribed verbatim. Two authors (AK and KBZ) independently coded interview transcripts. The coders underwent intercoder agreement exercises prior to and during the coding process. A full transcript was coded by both coders and intercoder agreement was formally assessed based on the use of the same categories (sub-themes) for each passage of text; there was a high level of agreement (*κ* ≥ 0.9). Any coding disagreements were discussed between the coders, and those unable to be resolved were arbitrated by a third coder (JRE or RL). The sampling and interview processes continued until thematic saturation was achieved [41].

### Analyses

Demographic, clinical, and survey data (SF-36 and IVI) are summarized and reported as medians and ranges. The Mann-Whitney U test was used to for inter-group comparisons. Scores on the IVI were transformed using published Rasch-calibrated values [42] since there were too few participants to precisely calibrate items for this study sample. The correlation of visual acuity and contrast sensitivity (on log scales) with IVI scores (on logit scale) was assessed by Spearman correlation since a non-linear but monotonic relationship was hypothesized. Pearson correlations were used to analyze correlations between various IVI domain scores.

Qualitative analyses were conducted using an inductive approach without predetermined codes. Codes were combined and then winnowed to address the research question, and resulting codes were binned into common categories, which were aggregated into overarching themes. The process was carried out in an iterative fashion, starting before the completion of interviews so that thematic analysis and eventually thematic saturation was achieved, signaling an adequate sample size [41]. A matrix analysis was performed in order to examine each VR-QOL theme and category by cause of severe PFL (RP or glaucoma) [43]. Coding and qualitative analyses were performed using MAXQDA Plus 2018 (VERBI Software, Berlin, Germany).

## Results

Sociodemographic characteristics of participants are presented in Table 1 stratified by cause of PFL. Additional details on each participant are presented in Supplementary Tables 1a and 1b. Overall, 19 participants (51%) were female, median age was 61 years old (range, 18–82), and duration of disease was 24 years (range, 1–61). Table 2 contains clinical characteristics and survey response data stratified by cause of PFL. Participants had a wide range of BCVA (20/20–20/800) and contrast sensitivity (0.00–1.65 log contrast sensitivity), while visual fields were uniformly constricted (RP: 4–20^○^; glaucoma: mean deviation − 31.4 to −9.9 dB).

**Table 1:** Participant demographic characteristics

	Glaucoma (*n* = 18)	Retinitis Pigmentosa (*n* = 19)
**Age (years)**		
*Median*	73	53
*Range*	53–82	18–74
**Gender n (%)**		
*Female*	44%	58%
**Race**		
*White*	47%	84%
*Asian*	6%	0%
*Middle Eastern*	11%	11%
*African American*	33%	5%
**Education**		
*Some High School*	0%	5%
*High School*	14%	16%
*Some College*	43%	11%
*College Degree*	0%	42%
*Advanced Degree*	43%	26%
**Employment**		
*Unemployed*	0%	16%
*Retired*	69%	11%
*Homemaker*	0%	5%
*Student*	0%	11%
*Part Time*	19%	32%
*Full Time*	13%	26%
**Living Situation**		
*Alone*	24%	16%
*With partner/spouse*	65%	74%
*With other family/friends*	12%	11%

**Table 2:** Participant clinical characteristics

	Glaucoma (***n*** = 18)	Retinitis Pigmentosa (***n*** = 19)
**Visual acuity (better eye)**		
*Median*	20/27.5	20/40
*Range*	20/20–20/150	20/20–20/800
**Visual field**^**a**^		
*Median*	−16.05 dB	10°
*Range*	−31.42- -9.86 dB	4–20º
**Pelli-Robson contrast sensitivity (log contrast sensitivity)**		
*Median*	1.1	1.2
*Range*	0.5–1.5	0.00–1.7
**Duration of disease (years)**		
*Median*	10	26
*Range*	1–22	3–61
**Other ocular comorbidity**		
*Cataract*	89%	58%
*Usher Syndrome*	0%	26%
*Other*	6%	11%
**Medical comorbidity**		
Yes	94%	53%
*Depression or anxiety*	22%	32%
*Hypertension*	61%	0%
*Heart Disease*	22%	0%
*Stroke*	11%	0%
*Diabetes*	28%	0%
*Lung Disease*	22%	0%
*Arthritis*	39%	0%
*Other*	28%	21%
**Low vision device use**		
*Yes*	50%	84%
**Low vision rehabilitation experience**		
*Yes*	39%	63%

*dB: decibel, RP* retinitis pigmentosa

^a^ For glaucoma: visual field mean deviation in decibels; for RP widest diameter of III4e isopter on Goldmann visual field test

Scores for each of the eight domains on the SF-36 are reported in Table 3 stratified by cause of PFL. There was variation in scores across IVI domains, as shown in Table 3. Sores on the IVI reading (*p* = 0.47) and mobility (*p* = 0.25) domains were not significantly different between participants with glaucoma and RP, but those with RP had lower emotional well-being scores (*p* = 0.01). Scores on the IVI were significantly correlated with better-eye but not worse-eye BCVA across all 3 domains in participants with glaucoma. IVI scores were not significantly correlated with better- or worse-eye BCVA in participants with RP. Contrast sensitivity was significantly correlated with the reading and emotional well-being domains in glaucoma and only the emotional well-being domain in RP (Table 4).

**Table 3:** Patient Reported Outcome Measure domain scores. Median (interquartile range) scores on each domain of the RAND 36-Item Health Survey (SF-36) and the Impact of Vision Impairment questionnaire

	Glaucoma (***n*** = 18)	Retinitis Pigmentosa (***n*** = 19)
**SF-36**^**a**^		
Physical functioning	80 (55–95)	89 (77–97)
Role limitation due to physical health	100 (25–100)	100 (69–100)
Role limitation due to emotional health	100 (100–100)	100 (100–100)
Energy/fatigue	70 (49–80)	66 (49–77)
Emotional well-being	84 (70–89)	84 (72–92)
Social functioning	100 (72–100)	94 (75–100)
Pain	75 (57–90)	90 (68–100)
General health	73 (58–80)	75 (69–80)
**IVI**^**b**^		
Reading	1.2 (− 0.5–1.8)	0.7 (0.0–1.1)
Mobility	0.5 (−0.5–0.8)	− 0.2 (− 0.7–0.4)
Well-being	0.9 (0.5–1.3)	0.1 (− 0.8–0.5)

*IVI* Impact of Vision Impairment, *SF-36* RAND 36-item Health Survey

^a^ Scores range from 0 to 100

^b^ Scores on interval logit scale

**Table 4:** Correlations between IVI scores and clinical vision measures

	Visual acuity better-eye^**a**^	Visual acuity worse-eye^**a**^	Pelli-Robson score binocular^**b**^	IVI reading	IVI mobility
***Retinitis pigmentosa***					
IVI Reading	−0.01	−0.10	0.10	–	–
IVI Mobility	0.22	0.12	−0.36	0.5*	–
IVI Well-Being	0.21	0.27	−0.46*	0.4	0.8**
***Glaucoma***					
IVI Reading	−0.66**	−0.15	0.60**	–	–
IVI Mobility	−0.47*	− 0.37	0.37	0.8**	–
IVI Well-Being	−0.61**	−0.11	0.48*	0.8**	0.7*

*IVI* Impact of Vision Impairment, *logCS* log contrast sensitivity, *logMAR* logarithm of minimum angle of resolution

^a^ On logarithm of minimum angle of resolution (logMAR) scale

^b^ On log contrast sensitivity (logCS) scale

*P < 0.05, **P < 0.01

**Fig. 1:**
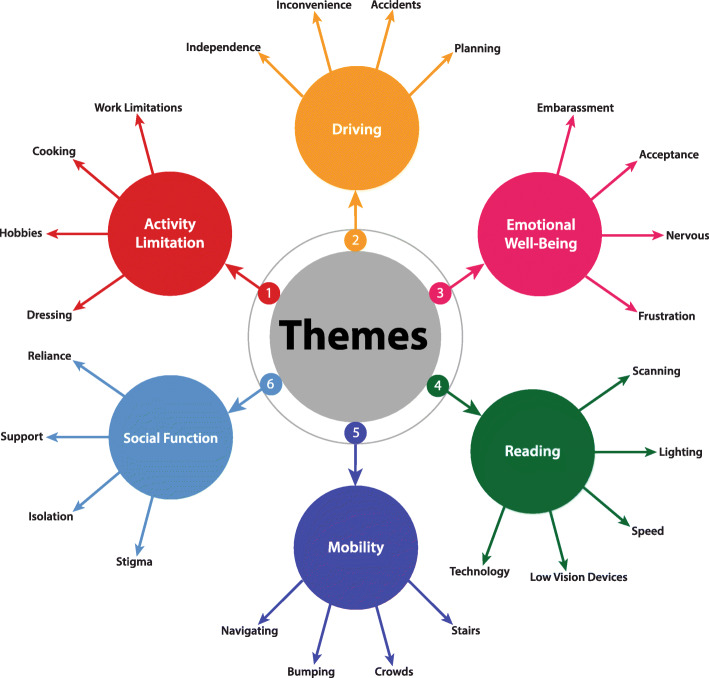
Vision-related quality of life themes. The six quality of life themes that emerged in this study were activity limitation, driving, emotional well-being, mobility, reading, and social function. Representative sub-themes are illustrated

Thematic analysis identified overarching VR-QOL themes. Figure 1 illustrates the 6 major themes that were identified: activity limitations, driving, emotional well-being, reading, mobility, and social function.

### Activity limitations

All participants cited daily activities that were made difficult because of their vision impairment. Participants also tended to avoid activities that required them to be around a large crowd of people or in unfamiliar places like airports, concert venues, or shopping malls. Most stated that movie theaters were too dark and the screens too large for their limited peripheral vision and some noted that large computer monitors were challenging for the same reason.

*One of the main things that bothered me with other people my age were going and doing sports, video games, different various outdoor activities, and it’s just much more difficult even though I have very, very accommodating, kindhearted friends who try to work with me. (22 year-old male; RP).*

Some participants mentioned having to make adjustments in their roles at work, sometimes due to safety concerns. Many had also given up visually demanding hobbies and sports that they had once enjoyed.

### Driving

Many participants discussed difficulties and dangers related to driving. Those who were no longer able to drive often felt isolated and limited in their ability to participate in work and social activities, and they discussed needing to rely on others. They consistently noted that this made them less independent.

*I have to be independent, and it's getting more and more of a concern, because I know my vision keeps getting worse and worse, so I don't know what I'm going to do when I get to the point where I can't drive. (82 year-old female; glaucoma)*

Some stated that if they could regain one function, it would be driving. Many relied on public transportation. Those who could afford ride-hailing services noted these services afforded greater independence. Others found that their inability to drive made them ineligible for certain jobs.

### Emotional well-being

Participants described feelings of frustration related to their limited vision. Some described needing to plan ahead and work at a slower pace; others were frustrated by stumbling and bumping into things. Many described frustration due to the need to wait several minutes to allow their vision to adjust following a lighting transition. Embarrassment and anxiety were also common. Participants described feeling embarrassed when they bumped into a person or object, or when people instinctively moved out of the way for them. For many, severe PFL meant that they could be easily startled by people or objects outside of their central vision, and this provoked anxiety.

*I try to describe to people that I'm kind of like a horse with blinders on...If I haven't heard them or know someone's there, I'm easily startled with things. I think partly that being startled comes from getting accustomed to the fact that things have to get so close to you before you see them. (61 year-old female, RP)*

Sadness, anger, and even clinical depression, occurred in response to losing independence. Some expressed feelings of helplessness, wondering why this happened to them, or why their disease had progressed faster than others’.

*My self-esteem went down. I got depressed. I didn't want to accept it. It's embarrassing. It's like you can't have fun anymore. It's like your eyesight is so important. I would rather have my arm or my leg cut off. I just want my sight to see the beauty of the world. (53 year-old male; glaucoma)*

Several older participants regretted not fully taken advantage of better vision earlier in life. Notwithstanding, many expressed gratitude for what they are able to do in spite of severe PFL.

### Reading

All participants experienced difficulties related to reading. Difficulty reading made some tasks, such as using the dials on a stove, potentially dangerous. Many noted they did not read for pleasure as much as they used to, often due to the small-size of print. Reading menus in dimly lit restaurants was particularly challenging, causing some to prepare in advance by reading the menu online, while others used adaptive devices. They also commonly expressed frustration due to progressive loss in reading speed as their disease worsened.

*I've always been a reader. I just can't do it anymore. I just can't go into a library and browse or a bookstore… The casual reading, the casual access to that kind of detail is gone. (72-year-old male; glaucoma)*

Some found that available assistive devices for reading were challenging to use due to PFL. For example, closed-circuit televisions and magnifiers made words so large that only part of the word was visible, which necessitated more scanning. Some accessibility features on smartphones, tablets, and computers, such as easily adjustable font size and contrast, were widely used and deemed helpful. However, many noted that severe PFL made it so difficult to navigate continuous text that they missed or had to re-read lines. Several explained that larger computer monitors and larger print is not necessarily easier for someone with severe PFL, and may even impose a greater challenge.

*If things print bigger, that doesn't help me at all...I don't need big letters…nothing that [low vision specialists] were talking about was helpful for me, so I didn't really want to go back…My low vision is so different than their low vision, and the things that they need. (74 year-old female; RP)*

### Mobility

A primary concern for all participants was safe mobility. Many described environments that were particularly challenging, including those that were unfamiliar, dark, or crowded. They noted that obstacles such as bicycles, strollers, and small children were often difficult to detect. Street crossings were difficult and often dangerous, as it was challenging to see oncoming vehicles, especially those turning corners, with PFL. Several had been hit by cars and had sustained serious injuries.

Unfamiliar places were challenging because of unknown obstacles. Participants frequently bumped into other people and objects like cupboards, shelves, street poles, tables, and “wet floor” signs located below eye level.

*…I don't like to be in crowds or crowded areas where there's people coming from my sides and stuff, for obvious reasons, because I'll run into them. And so, I kind of avoid those kind of settings unless I'm with somebody and I can follow them…I keep my head on a swivel and I'm constantly looking around. (48 year-old male; RP)*

Several cited bumping into furniture in their own home as an indicator of disease progression. Tripping and falling was a frequent concern. They noted tripping over uneven bricks or sidewalks, clutter around the house, and rugs. Tripping or falling on stairs was also common, with descending stairs considered more hazardous than ascending stairs. Due to severe PFL, participants continuously had to shift their gaze to avoid injury.

*My environment is so small. It’s like I’m sort of like in a closet. When I look out, I can’t see say more than about 10 ft, or 12 ft ahead of me. For example, if a bus is coming, I don’t see the bus until it’s about two car lengths in front of me…..(81 year-old female; glaucoma).*

### Social function

Close connections were sometimes challenging to maintain but also helped participants to cope with their vision impairment. Often these close social connections were with spouses or partners who often functioned as drivers, sighted guides, and advocates, even as other social functions with friends, family, and acquaintances became more difficult.

*I think I have had a wonderful life, and in great part because I had a spouse who didn't make me feel inadequate, and helped in the places that I needed help, and still helped me to feel important and needed... (74 year-old female; RP)*

Many worried that if their vision worsened they would become more dependent and that this may impact their interpersonal relationships. Existing social relationships frequently suffered along with the inability to drive and do activities that participants previously enjoyed with friends or family. Moreover, difficulty recognizing faces and making eye contact were barriers to meeting people and building new relationships.

### Comparing RP and glaucoma

A matrix analysis was conducted to compare VR-QOL concerns across themes and categories among those with RP and glaucoma (Supplementary Table 2). Many concerns were similar between participants from the two groups, though there were some notable differences that may be attributable to differences in the underlying diseases, the demographics of affected individuals (e.g., age), or a combination thereof. Differences notwithstanding, VR-QOL themes and categories were largely consistent across participants with both causes of severe PFL.

## Discussion

The objective of this study was to understand the day-to-day impact of severe PFL on the lives of individuals with RP and glaucoma. Participants expressed concerns related to six overarching VR-QOL themes: 1) limitations in vision-dependent activities, 2) difficulty reading, 3) challenges related to driving and independence, 4) limitations in safe mobility, 5) emotional concerns related to vision loss, and 6) impact on social function. In contrast to many prior qualitative studies, this study adopted a functional perspective, considering the impact of severe PFL, rather than the effect of a specific disease state on VR-QOL. This approach may provide data that is more relevant to rehabilitation, since low vision rehabilitation seeks to address the impact of vision loss irrespective of the underlying cause. While many of the overarching VR-QOL themes identified in this study have been previously reported [44–46], the *content* of these themes was distinct among participants in this study. As illustrated, the categories/sub-themes (Fig. 1) and their content (Supplementary Table 2) point to the specific and distinct impact of severe PFL on these six VR-QOL domains. Since the impact of severe PFL on VR-QOL has not been widely studied, these findings are important to the development of a PRO that is relevant to the experiences of individuals with severe PFL, which will fill a critical gap and may facilitate future research to identify evidence-based vision rehabilitation solutions for this population.

This study builds on prior quantitative [8, 15, 47–50] and qualitative [33–35, 51] investigations into the lived experiences of individuals with RP and glaucoma. Quantitative investigations have identified reading, driving, mobility, lighting, and facial recognition as commonly affected tasks in RP, while those with glaucoma experienced impacted mobility, activity limitations, visual symptoms, and socioemotional well-being. Most prior studies used generic vision- or health-related QOL measures, though these instruments were not developed around the experiences of individuals with severe PFL. Additionally, purely quantitative studies may not adequately capture the experience of individuals living with a disease state since they are limited by the content of extant measures [52]. In fact, the U.S. Food and Drug Administration guidance on PRO development indicates that population- and condition-specific qualitative studies are needed to ensure an instrument’s content validity [53].

Prior qualitative investigations have not adequately quantified the extent of visual field loss in the study sample or did not limit inclusion criteria to those with severe PFL, though doing so is important for determining relevant VR-QOL concerns to develop a PRO for this population that is likely be responsive to change following an intervention or progression of disease. In developing content for the National Eye Institute Visual Functioning Questionnaire (NEI-VFQ), Mangione et al. performed 26 condition-specific focus groups, eight of which were among individuals with glaucoma and two with low vision [44]. Both sets of focus groups included heterogeneous groups of patients that were well-suited to the development of a general VR-QOL instrument. For example, those with glaucoma had different levels of disease severity and the low vision groups contained participants with various causes of low vision. That study identified seven key “problems”: general vision-related concerns, mobility, reading, social relations and activities, home activities, self-care, and personal finances. There is a high degree of agreement in the overarching VR-QOL domains identified in this study and other qualitative VR-QOL instrument development projects, including the NEI-VFQ [44]. However, in contrast to the NEI-VFQ, the content generated in this study points to a need for novel items targeting functions such as safe street crossing, navigating crowds, obstacle avoidance, orientation and mobility in familiar versus unfamiliar environments, and use of technologies like smartphones, tablets, and computers to accomplish recreational, daily living, and vocational tasks.

Massof and Stelmack have demonstrated that in order to detect the full effect of an intervention, a measure must be relevant to the functional impairments and goals of patients [54]. Although hundreds of instruments have been used to assess visual impairment [6, 55], none tests multiple functional domains and meets these criteria specifically for people with low vision due to severe PFL. In fact, in some commonly used low vision PROs, items sensitive to PFL have been removed to improve the instrument’s psychometric properties [26], and others have not been well-targeted to people with PFL [14].

This study also contributes to the literature on the differential impact of central and peripheral vision loss on daily functioning and VR-QOL. A multicenter study by Brown et al. found that there was considerable overlap in the functional complaints of patients with low vision due to various causes, but that those with diagnoses associated with PFL had some distinct impairments, particularly related to mobility [32]. Of note, that study did not associate diagnoses or functional impairments with magnitude of vision loss, though this might have helped to clarify further these relationships. One possibility is that differences in functional impairments due to central and peripheral vision loss may be related to the processing of visual information that is believed to occur via two distinct “cortical streams,” the ventral and dorsal stream [56, 57]. The ventral stream depends on central vision and processes object and pattern recognition, while the dorsal stream utilizes peripheral vision for spatial relations, perception of motion, and visually guided motor behavior.

In the current study, Rasch-calibrated IVI scores were calculated for each of the three IVI domains. Scores covered a wide range, indicating varying levels of functioning, which may be an indicator of good content validity in the present study since participants had varying levels of functioning and VR-QOL. Scores on IVI domains were moderately to strongly correlated with one another, except for the reading and well-being domains in participants with RP ; the reason for this finding is not clear, as reading was an important VR-QOL feature for participants with both glaucoma and RP. Since all participants in this study had a similar level of PFL it was not possible to correlate magnitude of PFL and IVI scores. However, it is notable that IVI scores in participants with RP were not significantly correlated with visual acuity, a result that is inconsistent with many prior low vision studies in which participants had predominantly central vision loss [42]. This finding further emphasizes the importance of developing and validating a PRO that is relevant to individuals with severe PFL, in whom VR-QOL may be less closely related to visual acuity. Conversely, in participants with glaucoma, visual acuity in the better-seeing eye was moderately correlated with scores in all three IVI domains, a finding that suggests that the impact of PFL and/or visual acuity loss may affect VR-QOL differently across disease states.

Importantly, there were some key demographic and clinical differences between the two groups that should be considered when interpreting results. Those with RP were younger (median 53 vs 73 years-old), and had a longer duration of disease (median 26 vs 10 years) than those with glaucoma. A greater percentage of participants with RP also were white (84% vs 47%), had a college degree (68% vs 43%), and used low vision devices (84% vs 50%). Nonetheless, health-related quality of life scores on the SF-36 were similar between the two groups and a matrix analysis (Supplementary Table 2) demonstrated that, in general, participants with severe PFL due to RP and glaucoma had shared VR-QOL concerns. While IVI well-being scores were lower in participants with RP, this difference was not readily apparent in the qualitative interview data, which supported considerable emotional impact of both diseases.

The findings from this study will be used to develop a PRO measure, the LV-SCOPE Questionnaire, to assess functioning and VR-QOL in severe PFL. In the next phase of the LV-SCOPE project, survey items will be mapped to qualitative findings using a combination of existing items from validated PROs and newly written items. The intended meaning of survey items will be tested in cognitive interviews prior to the final study phase in which modern psychometric approaches (e.g., Rasch modeling) will be used to refine the survey structure and test its psychometric properties. The LV-SCOPE Questionnaire may ultimately enable clinicians and researchers to measure functioning and VR-QOL using an instrument that is optimally aligned with the lived experiences of individuals with severe PFL.

There were several limitations to this study. First, the VR-QOL and functional challenges described may not be generalizable to other populations with distinct environmental, cultural, or geographic characteristics. Populations in areas with greater or lesser access to urban amenities may have distinct experiences with mobility and driving and this may also be shaped by local driving regulations. While thematic saturation was attained with our study sample, it is plausible that additional themes could be expressed by a distinct sample with PFL. Additionally, some of the issues participants cited may have been due to other health problems or co-occurring eye conditions. Findings may also have been affected by interviewer and social desirability biases. There were also several key strengths to this study. Prior to data analysis, coders underwent an intercoder agreement exercise that showed a high-level of agreement. An inductive approach was used to allow codes and themes to emerge from the data until thematic saturation as achieved and a matrix analysis was conducted to compare RP and glaucoma. In addition, those with preserved central vision were intentionally oversampled in order to highlight issues most likely to be related to severe PFL.

## Conclusions

This study is unique in its focus on individuals with severe PFL, a group for whom there is a lack of evidence to guide PRO assessment and low vision rehabilitation practice. The analysis of qualitative data is a necessary step in PRO development in order to optimize an instrument’s content validity [53] and sensitivity to the effect of vision rehabilitation [54]. The LV-SCOPE development and validation project may ultimately improve the ability of clinicians to measure the effectiveness of vision rehabilitation for those with severe PFL, while permitting researchers to carry out transformative research in this area.

## Supplementary Information

**Additional file 1:** **Supplementary Table 1a.** Detailed Participant Characteristics. **Supplementary Table 1b.** Detailed Participant Characteristics. **Supplementary Table 2.** Matrix Analysis by Cause of Severe Peripheral Field Loss.

**Additional file 2:** **Supplemental Appendix.** Semi-Structured Interview Guide

## Data Availability

Data from this study are not publically available due to the nature of qualitative data.

## Abbreviations

BCVA: Best-corrected visual acuity
GVF: Goldmann Visual Field
IVI: Impact of Vision Impairment
logCS: Log contrast sensitivity
logMAR: Logarithm of minimum angle of resolution
LV-SCOPE: Low Vision Severely Constricted Peripheral Eyesight
OD: Right eye
OS: Left eye
OU: Both eyes
PFL: Peripheral field loss
PRO: Patient-reported outcome
RP: Retinitis pigmentosa
VR-QOL: Vision-related quality of life
