# Supplementary material for: Vision-related quality of life in adults with severe peripheral vision loss: a qualitative interview study
Source: J Patient Rep Outcomes. 2021 Jan 13;5:7. doi: 10.1186/s41687-020-00281-y (PMC7806695; doi:10.1186/s41687-020-00281-y)
Supplement: Supplementary file 1 — Additional file 1: Supplementary Table 1a. Detailed Participant Characteristics. Supplementary Table 1b. Detailed Participant Characteristics. Supplementary Table 2. Matrix Analysis by Cause of Severe Peripheral Field Loss. [file 41687_2020_281_MOESM1_ESM.docx]

| **Participant ID** | **Age (years)** | **Gender** | **Race/Ethnicity** | **Age at Diagnosis (years)** | **Age at Visual Impact (years)** | **Other Ocular Diagnoses** |
| --- | --- | --- | --- | --- | --- | --- |
| ***Retinitis pigmentosa*** | | | | | | |
| 1 | 62 | F | Caucasian | 26 | 35 | AMD |
| 2 | 49 | F | Caucasian | 24 | 23 | Usher |
| 3 | 64 | M | Caucasian | 20 | 14 | Usher |
| 4 | 56 | F | Caucasian | 26 | 12 | Usher |
| 5 | 36 | F | Caucasian | 2 | 5 | Usher, Cataract |
| 6 | 61 | F | Caucasian | 8 | 55 | Usher, Cataract |
| 7 | 28 | M | Caucasian | 3 | 13 | Cataract |
| 8 | 32 | M | Caucasian | 19 | 13 | Cataract |
| 9 | 22 | M | Middle Eastern | 17 | 16 | None |
| 10 | 38 | F | Caucasian | 12 | 16 | None |
| 11 | 61 | F | Caucasian | 16 | 40 | Cataract |
| 12 | 57 | M | Caucasian | 13 | 27 | Cataract |
| 13 | 53 | F | Caucasian | 24 | 20 | Cataract |
| 14 | 27 | M | African American | 5 | 19 | None |
| 15 | 48 | M | Caucasian | 48 | 16 | Cataract |
| 16 | 56 | M | Caucasian | 36 | 25 | Cataract, glaucoma |
| 17 | 74 | F | Caucasian | 24 | 13 | Cataract |
| 18 | 59 | F | Caucasian | 35 | 34 | Cataract |
| 19 | 18 | F | Middle Eastern | 12 | 15 | None |
| ***Glaucoma*** | | | | | | |
| 1 | 72 | M | African American | 48 | 65 | Cataract |
| 2 | 53 | F | Caucasian | 21 | 37 | None |
| 3 | 57 | M | African American | 51 | 46 | Cataract |
| 4 | 72 | M | Caucasian | 22 | 65 | Cataract |
| 5 | 53 | M | African American | 49 | 46 | AMD |
| 6 | 68 | M | Middle Eastern | 46 | 46 | Cataract |
| 7 | 81 | F | African American | 40 | 73 | Cataract |
| 8 | 75 | M | Middle Eastern | 74 | 74 | Cataract |
| 9 | 74 | F | African American | 68 | 68 | Cataract |
| 10 | 78 | F | Caucasian | 56 | 56 | Cataract |
| 11 | 76 | M | Asian | 71 | 71 | Cataract |
| 12 | 82 | F | Caucasian | 62 | 62 | Cataract |
| 13 | 61 | F | Caucasian | 45 | 45 | Cataract |
| 14 | 65 | F | Caucasian | 55 | 55 | Cataract |
| 15 | 60 | M | African American | 47 | 47 | Cataract |
| 16 | 78 | M | Caucasian | 63 | 63 | Cataract |
| 17 | 79 | M | Caucasian | 74 | 74 | Cataract |
| 18 | 76 | F | Caucasian | 66 | 66 | Cataract |

**Supplementary Table 1a.** **Detailed Participant Characteristics.**

AMD: age-related macular degeneration, F: female, M: male

**Supplementary Table 1b.** **Detailed Participant Characteristics.**

| **Participant ID** | **Visual Acuity (OD)^1^** | **Visual Acuity (OS)^1^** | **Contrast Sensitivity^2^** | **Visual Field (OD)^3^** | **Visual Field (OS)^3^** | **IVI Reading^4^** | **IVI Mobility^4^** | **IVI Emotional^4^** |
| --- | --- | --- | --- | --- | --- | --- | --- | --- |
| ***Retinitis pigmentosa*** | | | | | | | | |
| 1 | 20/200 | 20/250 | 0.75 | 15 | 20 | 1.11 | 1.64 | 1.75 |
| 2 | LP | 20/200 | 0.45 | 5 | 10 | 0.50 | 1.00 | 1.38 |
| 3 | 20/800 | 20/400 | 0.00 | 10 | 7 | 1.67 | 1.55 | 2.00 |
| 4 | 20/40 | 20/40 | 1.65 | 18 | 16 | 1.56 | 1.20 | 0.50 |
| 5 | 20/800 | HM | 0.00 | 8 | Unable | 2.83 | 2.91 | 2.88 |
| 6 | 20/50 | 20/40 | 1.65 | 10 | 12 | 2.11 | 0.82 | 0.88 |
| 7 | 20/800 | 20/600 | 0.00 | 5 | 5 | 2.11 | 2.36 | 2.00 |
| 8 | 20/30 | 20/60 | 1.50 | 19 | 15 | 2.11 | 1.60 | 2.00 |
| 9 | 20/30 | 20/30 | 1.05 | 13 | 15 | 1.89 | 1.36 | 1.25 |
| 10 | 20/125 | 20/100 | 0.75 | 7 | 7 | 1.11 | 0.82 | 0.50 |
| 11 | 20/40 | 20/40 | 1.35 | 18 | 18 | 2.22 | 1.09 | 1.00 |
| 12 | 20/400 | 20/400 | 0.45 | 9 | 11 | 1.89 | 2.00 | 1.63 |
| 13 | 20/50 | 20/800 | 0.75 | 10 | 6 | 1.33 | 1.09 | 1.63 |
| 14 | 20/40 | 20/40 | 1.65 | 4 | 4 | 1.56 | 0.45 | 0.00 |
| 15 | 20/40 | 20/40 | 1.35 | 10 | 11 | 2.67 | 2.18 | 2.50 |
| 16 | 20/20 | 20/20 | 1.20 | 5 | 5 | 1.11 | 1.27 | 2.13 |
| 17 | 20/40 | 20/40 | 1.20 | 5 | 5 | 2.67 | 2.36 | 2.63 |
| 18 | 20/150 | 20/30 | 1.35 | 14 | 11 | 2.22 | 1.45 | 2.00 |
| 19 | 20/30 | 20/25 | 1.65 | 8 | 8 | 1.78 | 1.73 | 0.50 |
| ***Glaucoma*** | | | | | | | | |
| 1 | LP | 20/60 | 0.90 |  | -24.9 | 0.22 | 0.00 | 1.75 |
| 2 | NLP | 20/25 | 1.35 |  | -16.05 | 2.78 | 2.09 | 2.25 |
| 3 | HM | 20/150 | 0.45 |  | -24.34 | 0.78 | 1.09 | 1.63 |
| 4 | 20/40 | 20/30 | 1.05 | -10.4 | -10.36 | 1.33 | 2.00 | 1.50 |
| 5 | 20/50 | 20/70 | 1.05 | -24.37 | -24.05 | 0.11 | 0.36 | 0.00 |
| 6 | 20/20 | CF | 1.20 | -16.06 |  | 2.78 | 2.73 | 2.38 |
| 7 | 20/300 | 20/125 | 0.60 |  | -17.12 | 0.56 | 1.27 | 2.25 |
| 8 | 20/30 | 20/30 | 1.50 | -24.79 | -15.41 | 2.38 | 2.27 | 2.50 |
| 9 | 20/25 | 20/25 | 1.35 | -10.08 | -9.86 | 2.67 | 2.82 | 2.75 |
| 10 | LP | 20/20 | 1.20 |  | -14.67 | 2.44 | 2.00 | 2.88 |
| 11 | 20/20 | 20/40 | 1.05 | -13.56 | -26.81 | 2.67 | 2.55 | 2.63 |
| 12 | 20/20 | 20/30 | 1.35 | -15.13 | -19.03 | 1.78 | 2.09 | 2.13 |
| 13 | 20/20 | 20/600 | 1.05 | -27.64 | -26.79 | 1.00 | 0.73 | 2.13 |
| 14 | 20/60 | 20/25 | 1.35 | -16.72 | -17.17 | 2.67 | 1.82 | 2.75 |
| 15 | 20/30 | 20/30 | 0.75 | -31.42 | -32.10 | 2.44 | 2.27 | 2.38 |
| 16 | 20/1250 | 20/20 | 1.20 | -28.98 | -12.03 | 2.33 | 2.30 | 2.88 |
| 17 | 20/20 | 20/30 | 1.35 | -12.87 | -26.89 | 2.22 | 1.27 | 2.25 |
| 18 | 20/60 | 20/50 | 1.05 | -23.7 | -22.70 | 1.67 | 2.40 | 2.38 |

CF: count fingers, HM: hand motions, IVI: impact of vision impairment, LP: light perception, NLP: no light perception, OD: right eye, OS: left eye

**^1^** Snellen visual acuity

**^2^** Log contrast sensitivity from Pelli-Robson test

**^3^** Retinitis pigmentosa: widest horizontal extent of III4e isopter on Goldmann visual field; Glaucoma: mean deviation in decibels on 24-2 Humphrey visual field test

**Supplementary Table 2. Matrix Analysis by Cause of Severe Peripheral Field Loss.**

| **VR-QOL Theme and Category** | **Overall Thematic Breakdown** | |  |
| --- | --- | --- | --- |
|  | **Example Quotation** | |  |
| ACTIVITY LIMITATIONS | **RP** | **Glaucoma** |  |
| Work | “I was working four days a week with the doctor and bringing patients into rooms, doing their vitals and charting, and then I almost knocked over a patient…and that was when I decided that I wasn't safe for patients…that's when I became a phone nurse …And at least I still feel like I can contribute to society. I still have life left in me, and I could keep working.” (RP-6) | “General terms, loss of vision affects your total life. First of all, I had to stop working, so that was absolutely huge in my life. And just, you know, it's simple things in life, you don't understand or appreciate, even as putting toothpaste on a toothbrush. Something as simple as that is a challenge. So it's, like I said, it's an adjustment. You go through a huge adjustment in life. But through time, you know, you make the adjustment.”  (GL-3) |  |
| Cooking | “I have a bright green spatula, different color spatulas and stuff, so then I don't have to continue to look for everything…I felt like I was always looking for stuff. So I've just been trying to buy more brighter colored items…I felt like, if I put the spatula by the burger in the pan, the spatula's not gonna melt. So then I can just look at the end of the green spatula, and right there is my burger.” (RP-10) | “I love to cook and it's not that I can't cook but now it's very different. It used to be took the knife and you just cut everything up on the cutting board and threw it in the pan and you could cook. I can't do that anymore because I am most likely to cut my finger. Now I use scissors to cut everything”. (GL-13) |  |
| Hobbies | “But to not be able to get up in the morning and go on a run because it's dark and I can't see as well …I can go down on the treadmill at five, but it's clearly not as interesting as being out somewhere running in the morning. Again, it's sad, and it's disheartening. But I really do try to think a lot about the fact that I'm still able to run, and I still can do lots of things that many other people can't.” (RP-11) | “It's limited my physical activity. I hesitate to do things I used to do. Actually, I can't, as well. I can't play tennis much anymore. I can't tell exactly where the ball is going to come. That's been difficult. I don't tend to want to go out and do things as much as I used to. That's the biggest thing because it feels hard. It's difficult.” GL-17)  “I've always had a little model railroad somewhere or structures for model railroads and clubs and it's kind of a craft hobby, but it means working with small things and details. I just don't have it. Crossword puzzles. I mean, I love to do crossword puzzles, but again, what I've had to do is get a metal clipboard and…Put it on the clipboard and I can't work with it down. It's too far away.” (GL-4) |  |
| DRIVING | **RP** | **Glaucoma** |  |
| Independence | “I really miss that independence. Just being able to get in the car. And I'll tell you something. I was telling my wife, sometimes I'll have dreams occasionally to think what would it be if I could get in the car and be able to do things again? That would be a big thing.” (RP-3) | “That experience was overwhelming, to be honest with you… Far as transportation, just your day-to-day life, if you're used to cooking for yourself, your independence, huge. Huge. I mean, just being yourself. Recognizing people is always a big ... I don't recognize people anymore.” (GL-3) |  |
| Accidents | “Before I knew what my diagnosis was, I was having accidents…And then when I found out that issues was there, I was much better. I probably drove probably five more years than I should have before I gave it up” (RP-3) | “I got in an accident because it was a blind spot, you know, and I looked I didn't see a car right there, but it was a blind spot.” (GL-5) |  |
| EMOTIONAL WELL-BEING | **RP** | **Glaucoma** |  |
| Embarrassment | “Well the first time at the mall it was very humbling. I felt like I was a spectacle, felt like I was being watched and kind of embarrassed. I navigated well in the mall part, but I noticed when I went into stores, that they would have displays that jutted out or like racks that were really close, and then I had trouble in those areas.” (RP-6) | “My self-esteem went down. I got depressed. I didn't want to accept it. It's embarrassing. It's like you can't have fun anymore. It's like your eyesight is so important. I would rather have my arm or my leg cut off. I just want my sight to see the beauty of the world.” (GL-5) |  |
| Acceptance | “Basically kind of going through the various stages of the grieving process. What are they again? Anger, not necessarily in this order, but denial, anger, depression, bargaining, and then acceptance. Like I said, not that it all goes in that order, and sometimes you can, as your vision continually decreases, you can go back through some of those stages again. I don't think we ever get completely to the acceptance stage and stay there.” (RP-2) | “Accepting and liking it are two different things. I don't like it at all… If you don't accept it, it's not going to get you anywhere. Being mad for the rest of my life isn't going to to help me. So, you just do what you have to do and do what you can do, and reach out…There are people out there that will help you if you let them. And it's easier for me if I let them do it instead of making a fool of myself and trying to do it myself.” (GL-18) |  |
| Nervous | “My brother and sister got the same vision, so it's like, who's going to be there for us, you know what I mean? At the end of the day, I know we came into this world alone, but we was always raised close family-oriented, I'm saying I am my brother's keeper type, so when my vision gone, I can just imagine how theirs is going to be, you know, so I'm like, man, and that's what scares me the most...And I don't even got kids, but I do want kids. I just don't my kids to have to go through things like this.” (RP-14) | “I'm terrified of losing my vision. It's not an acute terror, it's just of all the things that could go wrong in my world personally, that would be the worst. Just do what makes sense and hope it doesn't happen.” (GL-4) |  |
| Frustration | “It's frustrating, but I think it's also I don't think our society does a particularly good job in terms of educating people towards a more inclusive society…I don't have total vision loss and so I think the cane in many people's minds indicates that I'm completely blind, whereas, it's technically a marker that someone has trouble seeing or navigating in some fashion. I think also because there's a wide variety of different kinds of low vision that RP I think is somewhat idiosyncratic because the things I need help with aren't the things most people would assume that I need help with.” (RP-16) | “An average day living with vision impairment…sometimes frustration, if you go somewhere. Like, for example, if I'm catching the bus, and I ask people what bus is this, and they look at me…or going to the bank, and I can't see where to sign my name, or ask them to fill out the slip for me, and I get some tellers that give me a hard time about that. What do you want me to do? I don't feel like I should have to explain to every person I meet that I'm legally blind. That part is frustrating.” (GL-3) |  |
| READING | **RP** | **Glaucoma** | |
| Lighting | “If I'm in a dimly lit space and I can't see to read the menu very well kind of a thing, then it's very difficult for me. I usually ask help or something from somebody.” (RP-7) | “Yes, I think what it is is the lighting. It's really bright lights, and that interferes a lot, and the fact that walking around a lot. I noticed that the blurriness tends to make me uneasy, a little wobbly, and I can't explain it that well, but it's primarily the lighting bothers me more than anything.” (GL-9) | |
| Low Vision Devices | “I used to be able to read a large print on normal font and stuff with reading glasses. Now I have to use my handheld magnifier, so there's a lot of things I learned to adapt to. Technology actually is making things a lot easier for me than it may have used to been. If I had the technology back then, I might've been able to accomplish a little bit more.” (RP-7) | “I wish that the magnifier was square instead of round so that it could cover more territory, if I just want to sit down for a minute and not, turn on the apparatus where I have the screen, that I could read more instead of being round, be square or oblong or something, that I could read a whole, you know, it's hard to read a line in a book and then go back down because I'm not sure if I'm on the same line or something.” (GL-18) | |
| Technology | “…a bigger screen? For me, that’s the last thing I need… I use my phone as a computer or my laptop as a relatively small screen. That having more real estate doesn't help me at all, it's just things get lost more easily. The phone is perfect because here at this point in my vision loss, it feels I can see the entire screen at once.” (RP-16) | “I increased the font size on the computer, and I've even made the cursor bigger. So yes to the extent that I could do that, I modified that. The other thing I've done is if there's a long article online, I'll download it into a file and increase the contrast.” (GL-4) | |
| MOBILITY | **RP** | **Glaucoma** | |
| Stairs | “Going down stairs is worse than going up stairs, because if you miss a step going up, or if you make an extra step going up, like stop, then you step up, it's just a, "Oh." But you miss a step going down, and you could be in real trouble. My father died because he fell down the steps. The basement steps.” (RP-17) | “Day to day difficulties. I don't have depth perception, so that's pretty tough. For me to step off the bus, I have to turn around, because I'm not sure how far I'm stepping down. Or to walk up the stairs. Stairs are horrifying to me at this point in my life, because I don't ever know where the first one's really at. So that's tough.” (GL-3) | |
| Crowds | “If there's a crowd of people walking down the street. We could be someplace where it's relatively quiet, and I go into the restroom, and then I come out and suddenly there's a whole bunch of people. Listening is one of our defenses or offenses or tricks or whatever we try to do to try to see what's going on in the environment. And when there's a crowd it's harder to negotiate. You lose those, say, individual clues of where things are.” (RP-13) | “In a crowd. That's where it really shows. When I was given the white cane, it was with the idea of trying to make sure that people identify that's what my problem is.” (GL-16) | |
| Bumping | “My other job that I had, there was a lot of stuff that was kind of low-lying around the shin area, and that's the worst spot for me to be looking, especially if there's stuff that's also at eye level. So you think about your head first and your shins second, but my shins always suffered, especially if somebody put something there and didn't tell me about it or I was just absent-minded at that moment and forgot that there's something there.” (RP-8) | “That's why I'm gonna have to start using it. I'm gonna have to start using it, because I'm so tired of kicking over wet floor signs, and with that cane it would tell me that's in front of me because I can't see it when I walk in the grocery store or somewhere and it's right there, and that's my blind spot, and I knock it over every time. Sometimes before I turn an aisle or I walk into somebody's, in back of them. I have to actually really turn and look before I even step.” (GL-5) | |
| Navigating | “I think that the hardest part is getting around areas I'm unfamiliar with…when I go outside, then it's a whole new thing and it's difficult to find where curbs are, or where things may be sticking out or...I don't have any furniture that sticks out that's a tripping hazard at my house, but if I go to someone else's they have coffee tables and things that I might not see.” (RP-6) | “But I get very frightened if I don't know where I am, and the cane makes me feel a little safer…It's just that my world is so small, that I could see…People can walk right up on me, because I can't see them. I have no peripheral vision. A person can come right up on me, and I don't know that they're there that kind of thing, or they're in back of me…Then I have my cane, and I'm really swinging it, really kind of wide, trying to protect myself.” (GL-7) | |
| SOCIAL FUNCTION | **RP** | **Glaucoma** | |
| Stigma | “If they can't walk a straight line without bumping into a wall, how could they do the job properly? We already know that blind people have a hard image to overcome, and I don't want to further that negative perception they have of blind people.” (RP-2) | Think about going into the store and not saying hi to somebody you know because you don't see their face. I know what I would think of people who didn't say hi to me. What the heck is wrong with them? I'm sure that there are times like that. There are times too when I run into you in the store. I try very hard to be very polite and say, "I'm sorry. Are you okay?" because they don't know. In the same breath, I also know they probably made a comment about the stupid woman who just ran into them without thinking. I'm not saying everybody does it but you can tell a lot of times by disgust of people what they think that you did it on purpose or that you're not considerate.” (GL-13) | |
| Close connections | “My son keeps me going and my husband of course. If it wasn't for them, I don't know where I would be. They encourage me to do things and I know he's only eight but "mommy I want you to play a game with me," and he'll adapt the game for us…He will move the pieces and I will remind whose turn it is.” (RP-5) | “Yeah. Absolutely. My husband does make fun of me but he's absolutely supportive of whatever… I've got a pretty good support network. And they're pretty sympathetic. We got people with bad knees. My knees are fine, my eyes are not fine so ... one cancels the other out.” (GL-14) | |
| Reliance | “I hate the idea of being a burden on somebody else. And let alone being dependent for just mobility or rather than being dependent for finances, mobility, and just being that 30 year old living in mom's basement.” (RP-9) | It becomes your life. I just can't do it, so that's what you do…but it sucks having to depend on other people. I do not like depending on other people. But when you have to, that's what you've gotta do.” (GL-3) | |

RP: retinitis pigmentosa, VR-QOL: vision-related quality of life
