# Supplementary material for: Vision-related quality of life in adults with severe peripheral vision loss: a qualitative interview study
Source: J Patient Rep Outcomes. 2021 Jan 13;5:7. doi: 10.1186/s41687-020-00281-y (PMC7806695; doi:10.1186/s41687-020-00281-y)
Supplement: Supplementary file 2 — Additional file 2: Supplemental Appendix. Semi-Structured Interview Guide [file 41687_2020_281_MOESM2_ESM.docx]

**Supplemental Appendix. Semi-Structured Interview Guide**

**A. Vision Impairment and Functioning**

1. Tell me about how vision loss affects your life. What is it like to have vision impairment?

2. Describe an average day, living with vision impairment.

3. Tell me about certain situations that are more difficult for you because of your vision.

4. Does your vision keep you from doing anything?

5. Tell me about any impacts (*effects)* your vision impairment has had on your independence.

6. Do you think your eye condition affects how other people perceive you?

7. Tell me about any other medical conditions you have and how they affect your day-to-day life.

8. Tell me about any recent changes in your vision

**B. Rehabilitation Experience**:

9. Have you made any changes around your home to help you get by with vision impairment?

10. Have you ever used any low vision devices?

11. Have you ever undergone any sort of low vision rehabilitation, for example with an occupational therapist, orientation and mobility specialist, or low vision therapist?

1. **Rehabilitation Goals**

12. Why have you (*decided to/decided not to*) pursue low vision treatments like assistive devices or rehabilitation in the past?

13. Tell me what you’d like to change about your eyes or your vision?
